# Evaluation of Multiple Impacts of Furfural Acetone on Nematodes In Vitro and Control Efficiency against Root-Knot Nematodes in Pots and Fields

**DOI:** 10.3390/antibiotics9090605

**Published:** 2020-09-15

**Authors:** Wanli Cheng, Xue Yang, Li Zeng, Dian Huang, Minmin Cai, Longyu Zheng, Ziniu Yu, Jibin Zhang

**Affiliations:** State Key Laboratory of Agricultural Microbiology and National Engineering Research Center of Microbial Pesticides, College of Life Science and Technology, Huazhong Agricultural University, Wuhan 430070, China; chengwanli@mail.hzau.edu.cn (W.C.); sj1501yx@webmail.hzau.edu.cn (X.Y.); zengli1993@webmail.hzau.edu.cn (L.Z.); huangdian@mail.hzau.edu.cn (D.H.); cmm114@mail.hzau.edu.cn (M.C.); ly.zheng@mail.hzau.edu.cn (L.Z.); yz41@mail.hzau.edu.cn (Z.Y.)

**Keywords:** furfural acetone, nematode, *Meloidogyne incognita*, biological control, reproduction, egg hatching, feeding, field experiment

## Abstract

Root-knot nematodes (RKNs) seriously endanger agricultural development and cause great economic losses worldwide. Natural product furfural acetone (FAc) is a promising nematicide with strong attractant and nematicidal activities, but baseline information about the impact of FAc on the reproduction, egg hatching, feeding, and growth of nematodes and its pest control efficiency in field are lacking. Here, the inhibition effects of FAc on nematodes in vitro and its RKN control efficiency in pot and field were investigated. FAc inhibited the egg hatching of *Meloidogyne incognita* by 91.7% at 200 mg/L after 2 days and suppressed the reproduction, feeding, and growth of *Caenorhabditis elegans* in vitro. In pot experiments, FAc in various dosages reduced the disease index of plant root significantly. In field experiments, FAc exhibited control effect on RKNs equivalent to commercial nematicides avermectin and metam sodium, with a reduction in disease index by 36.9% at a dose of 50 mg/plant. FAc also reduced the population density of RKNs in soil, with a reduction rate of 75.3% at the dose of 750 mg/m^2^. No adverse effect was detected on plant growth after FAc application. These results provide compelling evidence for development of FAc as an appropriate alternative for current nematicides.

## 1. Introduction

Plant parasitic nematodes (PPNs) are among the most serious plant parasites that cause plant diseases, and affect the growth of crops, resulting in economic losses [1,2]. Diseases caused by PPNs lead to more than 150 billion U.S. dollars in economic losses worldwide each year [3], more than half of which is caused by root-knot nematodes (RKNs) [4]. RKNs (*Meloidogyne* spp.) have been considered to be one of the most damaging nematode group in the world because they cause great global economic losses to the majority of cultivated plant species in subtropical and tropical regions [5]. Therefore, the presence of RKNs in cultivated crops must be controlled.

Multiple management methods—including crop rotations [6], crop interplants [7], development of nematode-resistant varieties [8], and application of chemical nematicides [9]—have been applied to control RKNs. Chemical nematicides are still the primary means to control RKNs because of their high efficacy, easy application, and rapid onset [10]. However, frequent and excessive use of chemical nematicides increases risks to ecological systems and even human health [11], which lead to restricted use or even total ban of the most effective chemical nematicides. Therefore, environmentally friendly and sustainable alternatives are urgently needed for RKN control.

Biological control is an environmentally acceptable means to reduce pest losses. A great number of microorganisms [12,13,14], plants [15,16], and their metabolites [17] have been reported to possess good nematicidal activity. Volatile organic compounds (VOCs) produced by microorganisms or plants have especially good prospects for agricultural applications because of their multiple effects and integrated control strategies against RKNs [18,19,20]. Furfural, a nematicidal VOC produced by several plants [18] with inhibition effects on the respiration and motility of *M. incognita* [21], reduces root galls in greenhouse and microplot experiments [22], and has been developed into the commercial nematicide CropGuard^®^ [21]. The product development of furfural and the study of its anti-nematode activity are good examples for the study of other VOCs.

Furfural acetone (FAc) is, also known as 4-(2-furyl)-3-buten-2-one. The molecular formula of FAc is C_8_H_8_O_2_, and molecular weight is 136.15 g/mol. FAc has been recognized as a flavoring agent and food additive by the Joint FAO/WHO Expert Committee on Food Additives [23]. FAc is a VOC produced by several microbes or plants [24,25], and has been considered as a promising alternative to currently available nematicides because of its nematicidal, fumigant, and attractant activities against *M. incognita* [24]. However, the effects of FAc on the reproduction, egg hatching, feeding, and growth of nematodes and control efficiency on RKNs in the field are still unknown.

The reproduction, feeding, and growth of *M. incognita* all almost occur in the root of plants and are difficult to directly observe in vitro under current experimental conditions. *Caenorhabditis elegans* is a model nematode to research the biological activity of natural compounds on PPNs and it is easy to breed and reproduce in the laboratory [26,27,28]. Therefore, *C.elegans* is a good model to test the inhibit effect of nematicides on PPNs.

This study mainly aimed to determine (i) the effects of FAc on the reproduction, egg hatching, feeding, and growth of nematodes; and (ii) the control efficiency of FAc on RKNs in pots and field. The results indicated that FAc inhibited the egg hatching of *M. incognita* and suppressed the reproduction, feeding, and growth of *C. elegans* in vitro, and exhibits control effect on RKNs similar to commercial chemical nematicides in field experiments.

## 2. Results

### 2.1. Effect of FAc on the Egg Hatching of M. incognita

The results indicated that FAc in various concentrations had adverse effect on the egg hatching of *M. incognita*, especially when the concentration was above 100 mg/L (Table 1). The egg hatching was remarkably inhibited at the concentration of 500 mg/L. the average numbers of worms hatched from a single egg mass after 2 and 4 days of 500 mg/L FAc treatment were only 2.44 and 2.67, respectively, whereas those from an egg mass treated with distilled water (control) were 47.58 and 74.31, respectively (Table 1). Hence, FAc has a strong inhibition effect on the egg hatching of *M. incognita*.

### 2.2. Effect of FAc on the Reproduction, Feeding, and Growth of C. elegans

**Figure 1:**
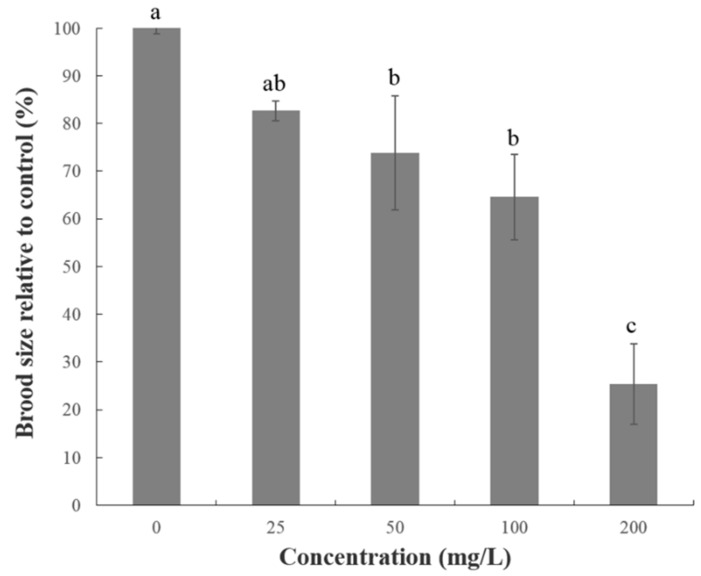
Reproduction assay of *C. elegans* immersed in FAc solution in various concentrations for 48 h. Data are shown as mean ± standard error (*n* = 3), and different lowercase letters indicate significant difference among treatments by LSD test (*p <* 0.05).

L4 worms were incubated with different concentrations of FAc at 20 °C for 48 h to assess the effect of FAc on the reproduction of *C. elegans*. As shown in Figure 1, FAc significantly affected the reproduction of *C. elegans*. FAc caused a consistent concentration-dependent decline in the brood size of a single worm, with an approximately 75% decrease in brood size after exposure to 200 mg/L FAc for 48 h.

In the presence of *Escherichia coli* OP50, *C. elegans* ingested food by rhythmic and sustained pharyngeal pumping. The pharyngeal pumping frequency of *C. elegans* decreased with increasing concentration of FAc. The pharyngeal pumping was reduced significantly when the concentration of FAc was above 25 mg/L after 48 h exposure, with an inhibition rate of 37.72% at the dose of 200 mg/L (Table 2).

**Figure 2:**
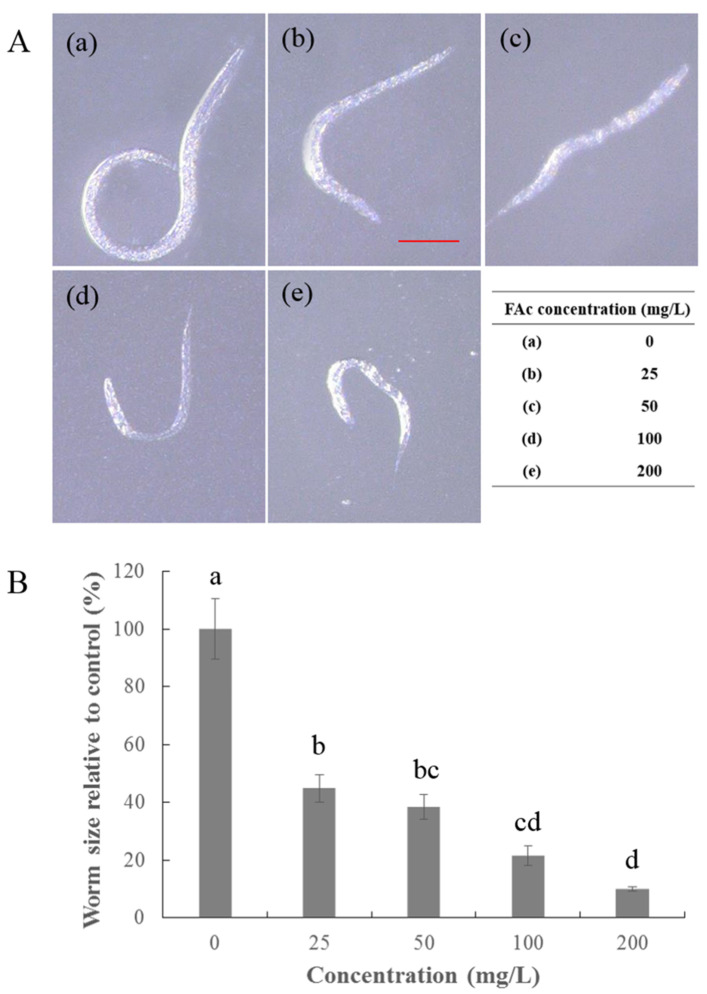
Growth assay of *C. elegans* treated with FAc. (**A**) (**a**–**e**) Wild-type L2 worms were cultured in gradient doses of FAc in a concentration range of 0–200 mg/L, incubated at 20 °C for 48 h, and photographed using a 3D digital microscope (Keyence VHX-6000, Keyence, Osaka, Japan). The scan bar is 100 μm. (**B**) The size of worms cultured in FAc as a percentage of the size of worms cultured in distilled water (control). Data are shown as mean ± standard error (*n* = 3), and different lowercase letters indicate significant difference among treatments by LSD test (*p <* 0.05).

FAc at various concentrations caused visible suppression of nematode growth with an obvious dose effect (Figure 2A), leading to approximately 55% decrease in size even at a low concentration of 25 mg/L (Figure 2B). Hence, FAc has a significant inhibition effect on the reproduction, feeding, and growth of *C. elegans*.

### 2.3. Efficiency of FAc against RKNs in Pots

**Figure 3:**
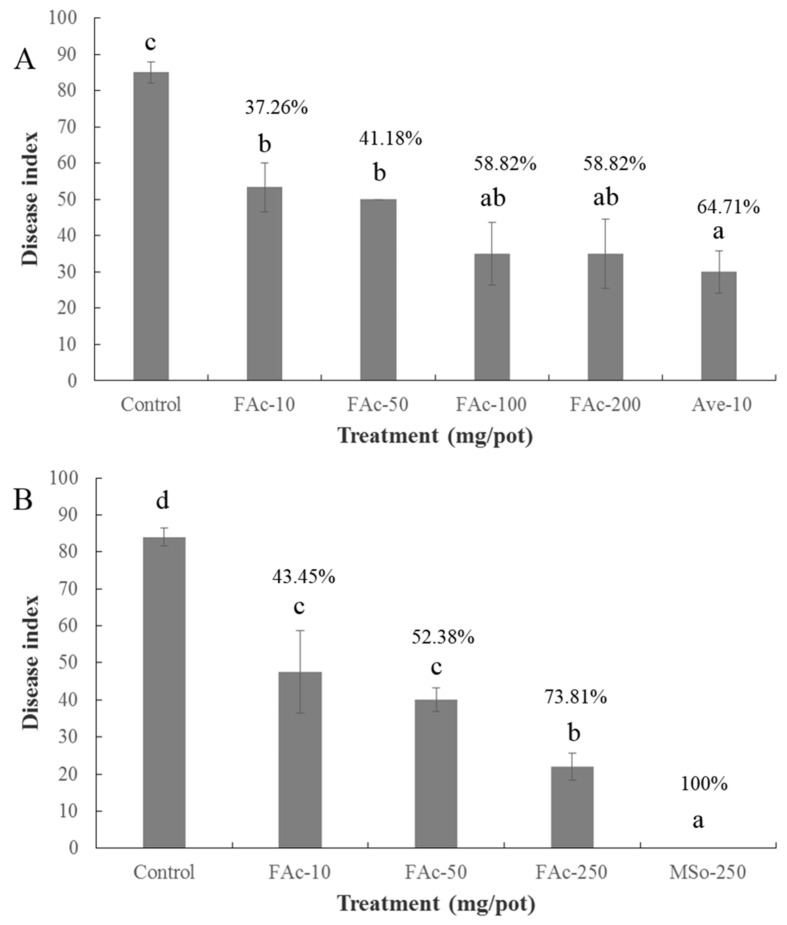
Control efficiency of FAc, avermectin (Ave), and metam sodium (MSo) on RKNs in pots. (**A**) Chemicals were irrigated into the rhizosphere of the plant. (**B**) chemicals were applied as fumigants. Data are shown as mean ± standard error (*n* = 5), and different lowercase letters indicate significant difference among treatments by LSD test (*p <* 0.05). The numbers above the lowercase letters indicate the control effect of each group compared with the control.

The disease index of the tomato root irrigated with FAc solution decreased significantly compared with that in the control group. The control effect of FAc at the dosage of 100 mg/pot was 58.82% compared with that of the control and showed no significant difference from that (64.71%) of avermectin at the dose of 10 mg/pot (Figure 3A). FAc also exerted good control effect on RKNs when applied as a fumigant, with control effects of 43.45%, 52.38%, and 73.81% at doses of 10, 50, and 250 mg/pot, respectively, which are lower than that (100%) of metam sodium (Figure 3B). In summary, FAc irrigated into the rhizosphere of the tomato plant or applied as a fumigant at different dosages have good control efficacy against RKNs in pots.

### 2.4. Efficiency of FAc against RKNs in the Field

**Figure 4:**
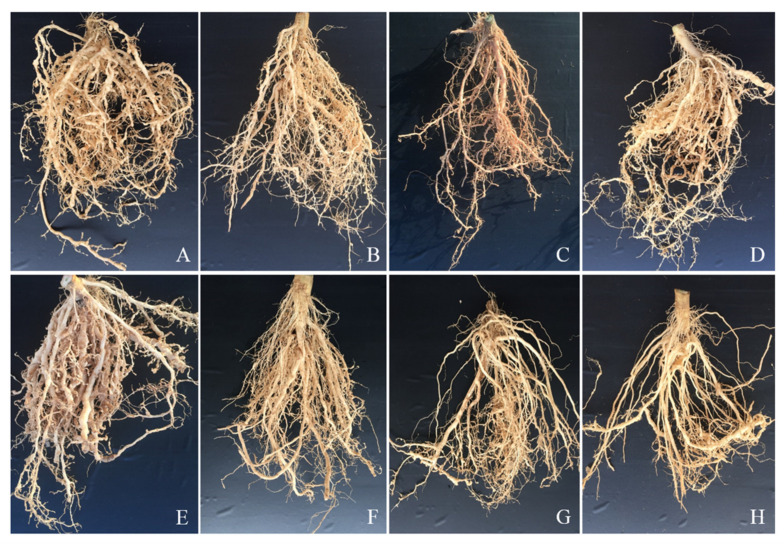
Root of tomato plants after treatment with (**A**,**E**) water (control), (**B**) 10 mg/plant FAc, (**C**) 50 mg/plant FAc, (**D**) 10 mg/plant avermectin, (**F**) 375 mg/m^2^ FAc, (**G**) 750 mg/m^2^ FAc, and (**H**) 750 mg/m^2^ metam sodium for 60 days in the field. (**A**–**D**): Chemicals were irrigated into the rhizosphere of the plant. (**E**–**H**): Chemicals were applied as fumigants.

FAc irrigated into the rhizosphere of the tomato plant or applied as a fumigant on RKNs in the pot experiments exhibited strong control efficacy, though FAc was less efficient than either avermectin or metam sodium. The RKN control effect was evaluated further in field experiments by irrigation of FAc into the rhizosphere of the tomato plant or application of it as a fumigant. RKNs infected and formed numerous large root galls in the control group, while fewer and smaller galls were observed on the roots after treatment with different dosages of FAc or commercial chemical nematicides through the two different application methods (Figure 4).

After irrigation of FAc into the rhizosphere of the tomato plants for 60 days in the field, the disease index in the FAc-treated groups decreased significantly compared with that in the control group, with a control effect of 30.2% at the dose of 10 mg/plant, which is equivalent to the control effect (29.5%) of 10 mg/plant avermectin (Table 3). When applied as a fumigant, FAc showed satisfactory nematode control activity, with control effects of 17.1% and 19.9% at doses of 375 and 750 mg/m^2^, respectively, close to the control effect (11.2%) of 750 mg/m^2^ metam sodium (Table 3). In addition, FAc applied at various dosages via two different methods reduced the population density of RKNs in the soil, with great reduction rates of 43.1%, 47.9%, 53.1%, and 75.3% at doses of 10 mg/plant, 50 mg/plant, 375 mg/m^2^, and 750 mg/m^2^, respectively (Table 3).

The growth of tomato plants was improved when FAc was applied as a fumigant, except that the height of plants treated with 375 mg/m^2^ FAc was slightly decreased compared with that of control. FAc irrigated into the rhizosphere of the tomato plant could improve the growth of plants at the dose of 10 mg/plant, and affected the growth of plants slightly at the dose of 50 mg/plant. However, no consistent and significant differences in the fresh weight on the ground, plant height, and stem thickness of tomato plant were found among the treatments (Table 4). It indicated that FAc has no adverse effect on plant growth in the field.

## 3. Discussion

Nematicides, such as avermectin [29], methyl bromide [30], and metam sodium [31], exhibit nematicidal or fumigant activity against RKNs, and most of them are toxic to animals and unfriendly to the environment [32,33,34]. Many RKNs have developed resistance to these nematicides that are continuously applied in the agricultural field for a long period worldwide [35]. Therefore, environmentally friendly and sustainable alternatives are urgently needed for RKN control. FAc has been reported to have attractant and nematicidal activities on RKNs [24]; in the present study, FAc inhibited the egg hatching of *M. incognita*, suppressed the reproduction, feeding, and growth of *C. elegans* (Figure 1 and Figure 2, Table 1 and Table 2). *C. elegans* is a model nematode to research the biological activity of natural compounds on PPNs [26,27,28]. Therefore, FAc may also suppress the reproduction, feeding, and growth of RKNs. As a VOC, FAc can spread quickly in the soil and then exert its inhibition effects on RKNs of various age stages via its multiple nematicidal activities. FAc is a natural product of several microorganisms and plants with nematicidal activity [24,25] and is relatively safe to humans because it can be used as a food additive in alcohol free drinks, ice, candies, gelatins, and other products of current life [23,36]. In addition, FAc can be chemically synthesized by using low-cost sugar cane residues [36], and can be degraded by soil microorganisms [37]. In the field experiment, FAc irrigated into the rhizosphere of the tomato plant or applied as a fumigant in the field could alleviate plant root-knot disease, exhibited similar control effect with commercial nematicides avermectin and metam sodium (Table 3), decreased the population density of RKNs in the soil (Table 3), and did not adversely affect the growth of tomato plants (Table 4). In conclusion, FAc possesses many advantages over current nematicides and has considerable potential to be developed as an appropriate alternative for current nematicides.

Many VOCs exhibit nematicidal, fumigant, or chemotactic activities against RKNs [19,20,24]. However, few works have investigated the effects of VOCs on the egg hatching, reproduction, feeding, and growth of nematodes. A previous study reported that FAc had strong nematicidal and attractant activities on RKNs [24], and the present work showed that FAc inhibited the egg hatching of *M. incognita*, and suppressed the reproduction, feeding, and growth of *C. elegans*. The multiple nematicidal activities of FAc made it possible to inhibit RKNs of different instars. The synergistic effect of multiple inhibition effects and attractant activities of FAc on nematodes may enhance its control efficiency on RKNs in agricultural production and achieve a strong RKN control effect in the field experiments (Table 3).

In the field experiments, FAc exerted similar nematode control effect to commercial nematicides when applied in the same dose via two different methods. However, the increasing of control effect on RKNs is not obvious with the rising of FAc dosage in the field experiments (Table 3). Low-dose FAc can achieve satisfactory nematode control effect in the field experiments and reduce the costs in agricultural applications. In addition, FAc irrigated into the rhizosphere of the tomato plant could reduce the disease index of plant root by 30.2% and 36.9% at doses of 10 and 50 mg/plant, respectively. However, when applied as a fumigant, FAc exhibited control effects of 17.1% and 19.9% at doses of 375 and 750 mg/m^2^ (150 and 300 mg/plant in average), respectively. Considering the cost and control efficiency against RKNs, FAc irrigated into the rhizosphere of the tomato plant could be a better application method compared with application as a fumigant. This study is the first to evaluate the control effect of FAc on RKNs in the field via two different application methods. The results lay a foundation for establishing methods of applications of subsequent VOCs in the field experiment. In contrast to commercial nematicides that can only be used as non-fumigants or fumigants, FAc can be used in the field via irrigation or fumigation because of its multiple impacts on RKNs. Therefore, a more reasonable application method of FAc can be selected in agricultural application. In conclusion, multiple application methods of FAc can enable farmers to select the more reasonable application method according to farmland and environment conditions.

VOCs can control RKNs or pathogens directly by killing, repelling, or inhibiting egg hatching [19,38,39]. Recent studies have also found that VOCs can promote plant growth and suppress pathogens by affecting the structure and abundance of microorganisms in the plant rhizosphere [40]. The control effect of FAc is lower than that of avermectin and metam sodium in pot experiments (Figure 3), but is similar to the same dose of avermectin and metam sodium when used in the field (Table 3). Hence, compared with experiments conducted in pot, FAc applied in the field may not only control RKNs by killing it or inhibiting its egg hatching directly, but also may have some other indirect mechanisms, such as affecting the microbial structure of the plant rhizosphere to enhance its inhibition efficiency. However, the specific mechanism of action is still unclear and deserves further research. 

In vitro, FAc inhibited the egg hatching of *M. incognita*, and suppressed the reproduction, feeding, and growth of *C. elegans*. FAc irrigated into the rhizosphere of the tomato plant or applied as a fumigant in the field showed that it could alleviate plant root-knot disease, exhibited similar control effect to commercial nematicides avermectin and metam sodium, decreased the population density of RKNs in the soil, and did not adversely affect the growth of tomato plants. Taken together, FAc has a considerable potential to be developed as an appropriate alternative to current nematicides.

## 4. Materials and Methods

### 4.1. Chemicals

Furfural acetone (purity ≥ 98%) was purchased from TCI (Tokyo Chemical Industry, Tokyo, Japan) and dissolved in distilled water or S medium at various concentrations to determine its effects on nematodes in vitro. Meanwhile, FAc was dissolved in dimethylsulfoxide to a concentration of 200 g/L and diluted to various concentrations with distilled water for pot and field experiments.

### 4.2. Nematodes

*Meloidogyne incognita* individuals were maintained on the roots of tomato (*Solanum lycopersicum*) grown in the greenhouse at 25 ± 1 °C. The egg masses of *M. incognita* were removed from the infected roots with needles, and washed three times in distilled water. The egg masses were placed in 24-well culture plates with distilled water at 20 °C for 3 days. Freshly hatched J2 juveniles were collected in a sterile tube. The fresh egg masses and J2 juveniles were used immediately.

Wild-type *C. elegans* (N2 Bristol strain) was provided by the Caenorhabditis Genetics Center and maintained at 20 °C on nematode growth media (NGM) seeded with *E. coli* OP50 [28].

### 4.3. Effect of FAc on the Egg Hatching of M. incognita

The influence of FAc on the egg hatching of *M. incognita* was determined by immersing the egg masses of *M. incognita* in FAc solutions in 96-well plates. Three egg masses and 200 μL of FAc solution dissolved in water were added to each well, and distilled water was used as the control. The number of J2s that hatched from the egg masses was counted under an inverted microscope (Olympus IX73, Olympus, Tokyo, Japan) after 2 and 4 days of exposure at 20 °C. The experiment was repeated three times, with three replicates at each concentration of FAc. The inhibition rate of *M. incognita* egg hatching was calculated by using the following equation.

Inhibition rate (%) = [(hatched worms per egg mass in control − hatched worms per egg mass in treatment)/hatched worms per egg mass in control] × 100.

### 4.4. Effect of FAc on the Reproduction of C. elegans

The effect of FAc on the reproduction of nematodes was performed in 96-well plates according to a described method [41]. Each well was added with 5 μL of *E. coli* OP50 cultured in S medium (OD_600_ = 2.0), a single L4 wild-type *C. elegans*, and 115 μL of FAc solution dissolved in S medium. The S medium instead of the FAc solution was used as control, and five wells were assayed for each concentration. The plates were placed at 20 °C for 48 h, and the number of eggs in each well was counted under an inverted microscope (Olympus IX73). The experiment was repeated three times, and the number of eggs produced by a single worm in each group was calculated and compared with the control.

### 4.5. Effect of FAc on Pharyngeal Pumping of C. elegans

The effect of FAc on the pharyngeal pumping of *C. elegans* was examined as a described method [42] to determine its influence on the feeding of nematodes. A total volume of 100 μL of FAc solution dissolved in water was added to 900 μL of NGM to final concentrations of 0, 25, 50, 100, and 200 mg/L. About 1 mL of the FAc-NGM mixture was pipetted into each well of a 24-well plate and left to dry overnight. Each well of the 24-well plate was added with 50 μL of the OP50 culture (OD_600_ = 0.6). The plates were left overnight to allow the OP50 lawn to grow at 20 °C. Six synchronized L4 wild-type *C. elegans* were placed in each well of the 24-well plate filled with *E. coli* seeded FAc-infused NGM at 20 °C. Pharyngeal pumping was counted at 24 and 48 h post-exposure. The worms were viewed under a 3D digital microscope (Keyence VHX-6000, Keyence, Osaka, Japan) and pumping rates were determined by visual observations. One pharyngeal pump was defined as a complete forward and backward movement of the grinder in the pharynx. The rate was counted for a period of 1 min and expressed in Hz. The experiment was repeated three times, with five replicates at each concentration of FAc.

### 4.6. Effect of FAc on the Growth of C. elegans

The growth assays for *C. elegans* were conducted according to a previously described method [41] with some modifications. Briefly, 10 μL of *E. coli* OP50 culture (OD_600_ = 2.0), 135 μL of FAc solution dissolved in S medium, and 5 μL of 20 L2 wild-type *C. elegans* were added to each well of 96-well plates. The S medium without FAc instead of the FAc solution was used as control. The plates were incubated at 20 °C on a rocking platform at a speed of 150 r/min for 48 h. After incubation, the worms of three wells treated with the same concentration of FAc were gently mixed and washed by distilled water three times. About 10 μL of the worms were pipetted onto a glass slide containing 15 mM sodium azide as an anesthetic. At least 10 nematodes in each concentration were photographed under a 3D digital microscope (Keyence VHX-6000). The size of these worms was calculated by using Image J1.33 software (NIH, Bethesda, MD, USA). The experiment was repeated three times, and the average size of the worms was plotted against that of the control group for comparison.

### 4.7. Control Efficacy of FAc against RKNs in Pots

The soil used in the pot experiment was collected from the RKN-infested field at Institute of Vegetables, Wuhan Academy of Agricultural Sciences (30.710038° N, 114.476825° E, Wuhan, Hubei, China), and mixed with organic matter at the ratio of 1:1. The seeds of tomato ZhongShu No.4 were surface disinfested in 2% sodium hypochlorite for 5 min, rinsed three times in distilled water, and incubated in organic matter for 2 weeks. Two-week-old plants of the same size were selected for pot experiments. Round plastic pots (18 × 18 × 12.5 cm) were filled with about 1 kg of the soil mixture. One two-week-old tomato seedling was transplanted into each pot and incubated in the greenhouse at 22–25 °C.

The two different application methods of FAc in the pot experiment were irrigation of FAc solution into the rhizosphere of the tomato plant and fumigation. When applied by irrigation, FAc was introduced into the rhizosphere soil of the plant by adding 50 mL of FAc solution 2 days after transplant. When used as a fumigant, 50 mL of the FAc solution was mixed with 1 kg of soil and covered with a plastic film for 10 days in the greenhouse at 25 °C. The plastic film was opened and deflated for 7 days. Each 1 kg of soil after fumigation was placed into round plastic pots and a single tomato seedling was transplanted into each pot.

When FAc was irrigated into the rhizosphere of plants, the experiment contained six treatments: FAc dissolved in water (10, 50, 100, and 200 mg/pot), 1.8% avermectin dissolved in water (10 mg/pot), or water alone (control). When FAc was used as a fumigant, the experiment comprised five treatments: FAc dissolved in water (10, 50, and 250 mg/pot), 45% metam sodium dissolved in water (250 mg/pot), or water alone (control). Each treatment had five replicates, and the disease severity of the plant root was assessed 60 days after transplant.

The disease severity of plant root was determined by rating the roots on each plant on a 0 to 10 scale based mainly on the types of roots galled by RKNs after 60 days. The scale 0 to 10 represented as follows: 0, no knots on roots; 1, few small knots but difficult to find; 2, small knots only and main root is clean; 3, some large knots visible and main roots is clean; 4, larger knots predominate and main roots is clean; 5, 50% of roots infected and knotting on parts of main roots; 6, knotting on main roots; 7, majority of main roots knotted; 8, all main roots knotted with few clean roots visible; 9, all roots severely knotted and plant usually dying; 10, all roots severely knotted and plant usually dead [43]. Disease index was calculated by using the following equation [44]. Nematode control effect was calculated by using the following equation [45].

Disease index = [Σ (the number of diseased plants with severity rating i × rating i)/(total number of plants × 10)] × 100.

Control effect (%) = [(disease index in control − disease index in treated)/disease index in control] × 100.

### 4.8. Control Efficacy of FAc against RKNs in Field

The field experiment was conducted in RKN-infested loamy soil at the Institute of Vegetables, Wuhan Academy of Agricultural Sciences, at a siteused to cultivate tomato plants before this batch of field experiments. The plots with tomato root-knot index of 7 were used to conduct field experiments. The tomato seedlings (ZhongShu No.4) of the same size grown in a seedling room for 15 days were used to transplant in the field experiments. FAc was irrigated into the rhizosphere of the plants or used as fumigant in the field. When applied by irrigation, 50 mL of FAc solution was irrigated into the rhizosphere of the plant 2 days after transplant; this experiment consisted of four treatments: FAc dissolved in water (10 and 50 mg/plant), 1.8% avermectin dissolved in water (10 mg/plant), or water alone (control). When FAc was used as a fumigant, two trenches with depth of 15 cm and length of 3.5 m were dug on the left and right sides of each plot (3.5 m in length and 1.6 m in width). A total of 5 L of FAc solution dissolved in water was spread evenly in the trench, and the soil was covered immediately. The soil moisture was adjusted to 70%. Each plot was covered by the plastic film and fumigated for 10 days. Tomato seedlings of the same size were transplanted to the plots 7 days after peeling off the film. This experiment involved four treatments: FAc dissolved in water (375 and 750 mg/m^2^), 45% metam sodium dissolved in water (750 mg/m^2^), and the same volume of distilled water (control). Each treatment consisted of three plots arranged randomly, and each plot included 14 plants. Compound fertilizer (China-Arab Chemical Fertilizer Co., Ltd., Qinhuangdao, China) was applied at the suggested dosage of 25 kg/mu in each plot before transplant to ensure normal plant growth. Disease severity, fresh weight on the ground, plant height, and stem thickness were assessed 60 days after transplant. The disease severity of plant roots and the control effect of chemicals were counted as above.

The tomato root soil of each plot was collected by picking a tomato from the front, middle, and back of each plot. The tomato root was pulled out, the soil attached to the root was collected, and three soil samples in the same plot were mixed. Afterward, 30 g of the soil sample in each plot was then selected to detect the population density of RKNs in the soil. Nematodes in the soil were collected into 90 mm Petri dishes by sugar centrifugation flotation [46]. Identification of RKN by its size and morphologlyl [47] from nematodes isolated from soil, and the number of RKNs was counted under an inverted microscope (Olympus, IX73). Reduction rate of RKNs (%) = [(number of RKNs in control − number of RKNs in treatment)/number of RKNs in control] × 100.

### 4.9. Statistical Analyses

All data were analyzed by SPSS (Statistical Package for the Social Sciences), version 22.0 software (SPSS, Chicago, IL, USA) and shown as mean ± standard error (SE) (*n* ≥ 3). Least significant difference (LSD) test was employed to test for significant differences among treatments at *p* = 0.05. Different lowercase letters indicate significant difference among treatments.

## Figures and Tables

**Table 1:** Effect of FAc on the egg hatching of *M. incognita*.

Concentration (mg/L)	2 Days		4 Days	
	Hatched Worms/Egg Mass	Inhibition Rate (%)	Hatched Worms/Egg Mass	Inhibition Rate (%)
0	47.6 ± 5.8 a ^1^	(0.0)	74.3 ± 9.3 a	(0.0)
50	40.1 ± 4.9 a	15.8	67.8 ± 10.1 a	8.8
100	16.9 ± 3.3 b	64.6	19.7 ± 5.3 b	73.5
200	4.0 ± 1.5 c	91.7	4.6 ± 1.5 b	93.8
500	2.4 ± 0.6 c	94.9	2.7 ± 0.7 b	96.4

^1^ Data are shown as mean ± standard error (*n* = 3), and different lowercase letters indicate significant difference among treatments by LSD test (*p <* 0.05).

**Table 2:** Effect of FAc on the food pharyngeal pumping of *C. elegans*.

Concentrations (mg/L)	Pumping ^1^ (Hz)	
	24 h	48 h
0	183.7 ± 2.6 a ^2^	175.1 ± 3.9 a
25	163.7 ± 8.8 ab	155.4 ± 2.7 b
50	164.3 ± 7.8 ab	147.9 ± 4.5 b
100	148.1 ± 7.8 b	135.5 ± 4.5 c
200	117.8 ± 2.9 c	109.1 ± 3.5 d

^1^ Pumping was counted for a period of 1 min and expressed as pumps/min (Hz). ^2^ Data are shown as mean ± standard error (*n* = 3), and different lowercase letters indicate significant difference among treatments by LSD test (*p <* 0.05).

**Table 3:** Suppression of RKNs by FAc on tomato plants in the field experiments.

Treatment	Disease Index	Control Effect (%)	Number of RKNs/30 g Soil	Reduction Rate of RKNs (%)
**Irrigation ^1^**				
Control ^2^	82.4 ± 2.2 b ^7^	(0.0)	432 ± 93 a	(0.0)
Fac ^3^ 10 mg/plant	57.5 ± 2.8 a	30.2	246 ± 62 a	43.1
FAc 50 mg/plant	52.3 ± 2.9 a	36.9	225 ± 97 a	47.9
Ave ^4^ 10 mg/plant	58.1 ± 3.0 a	29.5	401 ± 31 a	7.2
**Fumigant ^5^**				
Control	73.6 ± 2.6 b	(0.0)	1617 ± 1323 a	(0.0)
FAc 375 mg/m^2^	61.1 ± 3.8 a	17.1	759 ± 259 a	53.1
FAc 750 mg/m^2^	59.0 ± 2.6 a	19.9	400 ± 51 a	75.3
MSo ^6^ 750 mg/m^2^	65.4 ± 3.2 a	11.2	726 ± 314 a	55.1

^1^ Irrigation means the chemicals were irrigated into the rhizosphere of the plant. ^2^ Control means the same volume of water was applied to the field. ^3^ FAc means furfural acetone. ^4^ Ave means avermectin. ^5^ Fumigant means the chemicals were applied as fumigants. ^6^ MSo means metam sodium. ^7^ Data are shown as mean ± standard error (*n* = 3), and different lowercase letters indicate significant difference among treatments by LSD test (*p <* 0.05).

**Table 4:** Growth effect of FAc on tomato plants in the field experiments

Treatment	Fresh Weight on the Ground (g)	Plant Height (cm)	Stem Thickness (cm)
**Irrigation**			
Control	618 ± 78 a ^1^	89.5 ± 3.1 a	3.64 ± 0.13 a
FAc 10 mg/plant	737 ± 109 a	96.5 ± 3.7 a	3.76 ± 0.11 a
FAc 50 mg/plant	593 ± 72 a	85.3 ± 4.4 a	3.59 ± 0.14 a
Ave 10 mg/plant	655 ± 16 a	94.7 ± 2.8 a	3.72 ± 0.10 a
**Fumigant**			
Control	667 ± 52 a	101.0 ± 3.0 a	3.46 ± 0.09 a
FAc 375 mg/m^2^	724 ± 77 a	98.7 ± 3.4 a	3.70 ± 0.15 a
FAc 750 mg/m^2^	749 ± 44 a	105.4 ± 2.9 a	3.71 ± 0.11 a
MSo 750 mg/m^2^	821 ± 42 a	106.1 ± 2.7 a	3.63 ± 0.08 a

The means of Control, Irrigation, Fumigant, and abbreviations are the same as that in Table 3. ^1^ Data are shown as mean ± standard error (*n* = 3), and different lowercase letters indicate significant difference among treatments by LSD test (*p <* 0.05).
